# How to incorporate telemedicine in medical residency: A Brazilian experience in pediatric emergency

**DOI:** 10.1016/j.clinsp.2022.100162

**Published:** 2023-02-16

**Authors:** Rafael da Silva Giannasi Severini, Michelle Marcovici, Sylvia Costa Lima Farhat, Danielle Bivanco-Lima, Thomaz Bittencourt Couto, Ana Carolina Amarante, Katharina Reichmann Rodrigues, Danielle Saad Nemer Bou Ghosn, Cláudio Schvartsman

**Affiliations:** a Departamento Emergência, Instituto da Criança, Hospital das Clínicas, Faculdade de Medicina, Universidade de São Paulo, São Paulo, SP, Brazil; b Department of Public Health, Faculdade de Ciências Médicas da Santa Casa de São Paulo (FCMSP), São Paulo, SP, Brazil

**Keywords:** Telemedicine, Education, Residency, Pediatrics, Pediatric emergency

## Abstract

**Introduction:**

The exponential growth of telehealth services during the COVID-19 pandemic led to the implementation of a telemedicine care service in a tertiary university pediatric hospital. It brought the need to develop a training aimed at remote care within the pediatric emergency rotation program.

**Objective:**

To describe the implementation of a telemedicine training for pediatric residents and present the preliminary results.

**Methods:**

Descriptive prospective study (pre and post), with 40 resident physicians of the first year of pediatrics. Reaction Assessments were applied before and after training, in addition to a resident physician perception questionnaire at the end of the training.

**Results:**

There was a significant difference in the resident's perception of experience and safety after initial training. Most rated the proposal as good or excellent, considered teaching telemedicine relevant and that this experience contributed to their learning on the subject.

**Conclusion:**

This study describes an innovative proposal for training in telemedicine. The preliminary results were encouraging, demonstrating the program's potential in training future pediatricians.

## Highlights

• The exponential growth of telehealth services during the COVID-19 pandemic led to the implementation of a telemedicine care service in a tertiary university pediatric hospital. It brought the need to develop a training aimed at remote care within the pediatric emergency.
• This descriptive prospective study with 40 pediatric resident physicians describes an innovative proposal for training in telemedicine.
• The preliminary results were encouraging, demonstrating the program's potential in training future pediatricians.

## Introduction

Telemedicine is defined as “the remote delivery of health services and clinical information using technology of information and communication”.^1^^,^^2^ It has advantages over telephone communication, including audiovisual interaction made in real time between the doctor and the patient, under his direct visualization, from the patient's home, guaranteeing a higher quality approach.^1^, ^2^, ^3^

Telemedicine has expanded considerably in the last two decades, with more than half of hospitals in the United States using some form of telehealth.^1^^,^^2^^,^^4^ In Brazil, its use was definitively regulated in 2022, after the great expansion of services from 2020 onwards with the COVID-19 pandemic.^5^

## Telemedicine at our hospital

In April 2020, the Hospital Telemedicine Emergency Care Program (PATMHO ‒ “Pronto Atendimento em Telemedicina Hospitalar”, in Portuguese) started at the Integrated Pediatric Reference Emergency Center of our hospital.^6^

The implementation of a telemedicine service in an institution with medical residency allows creating a teaching environment for remote care. Given the exponential growth in the use of telemedicine due to the COVID-19 pandemic, associated with the implementation of PATMHO, the need arose to develop a teaching program in telepediatrics for the medical residency curriculum.

A systematic review from 2016, pre-COVID-19 pandemic, showed that there were already telehealth education programs, but with limited and incipient evidence.^7^ In 2020, a new review reaffirmed that there was still no consistent data on the educational integration of telehealth-related content, suggesting the need to establish a standardized curriculum, with training programs and basic skills.^8^ More recent studies show that formal education in telehealth increases residents' confidence and interest in the subject, improving physicians' preparation for clinical practice.^9^

The Cleveland Clinic residency program shared their experience in structuring a formal outpatient telemedicine rotation for Internal Medicine residents. The initial results showed good acceptance by residents, filling gaps in telehealth created by the COVID-19 pandemic and improvement of the program with criticism and suggestions from residents.^10^

Until this moment, we did not find studies that described a telehealth education program focused on the pediatric medical residency curriculum. Given the particularities of care in the pediatric population, we believe that the implementation of telecare teaching for pediatric residents will contribute to a more up-to-date training in the face of the new global demand for telehealth, changing their perception of the importance of this model of care also for the pediatric population.

The present study aims to describe the implementation of training for telemedicine care within the curriculum of pediatric residents and presents preliminary results in view of this novelty in the curriculum.

## Methods

This is a descriptive study reporting the implementation of telemedicine training in pediatric emergencies. It was performed in the pediatric emergency department of a public and tertiary university hospital. Only first-year pediatric residents during their passage through the pediatric emergencies stage were included. The study shows preliminary data of the first six months of the rotation.

The training is included in the pediatric emergencies rotation, so all residents go through it. They were invited to voluntarily participate in the project without any loss of learning if denied, with an online signature of the Informed Consent Form (ICF) to use the evaluation data used. Coordinators and attending physicians did not have information on who accepted to participate or not. None of the researchers of the project were involved in issuing the grades at the end of the rotation.

It is part of the thematic project approved by the Ethics and Research Committee of the Hospital. All study steps including forms and data collection methods were presented to the committee and approved.

### The telemedicine training

**Fig. 1:**
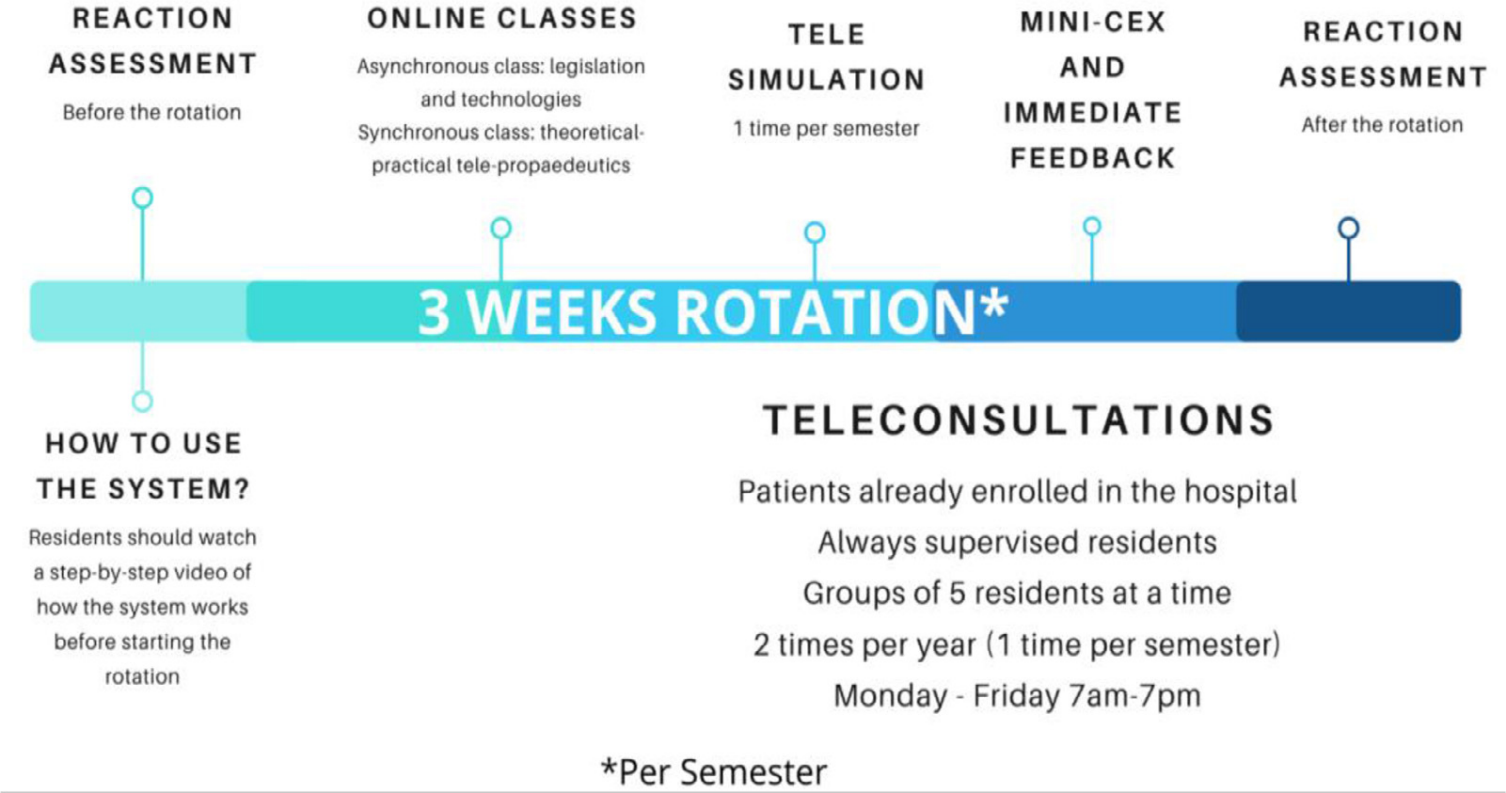
Telemedicine Rotation design proposal.

The pediatric emergencies rotation lasts six weeks, of which three are in the emergency department and three in the emergency room's rear ward, once a semester (1st semester from March to August and 2nd semester from September to February). First-year pediatric residents actively participate in the care of pediatric patients *via* telemedicine, under the face-to-face supervision of an attending physician, during the three-week in the emergency room. The service runs from Monday through Friday, 7 am to 7 pm. The design of the program is shown in Fig. 1.

### Educational objectives of telemedicine training

We have developed educational objectives for the telemedicine rotation, targeting skills development as part of a competency-based curriculum, inspired by CanMEDS 2015^11^:

- Understand the importance of telemedicine in the current conjuncture.
- Apply distance pediatric anamnesis and propaedeutics techniques.
- Recognize the limitations of telemedicine care.
- Identify signs of seriousness in teleconsultation that justify face-to-face care.
- Incorporating the use of telemedicine into clinical practice.
- Establish good communication with the patient and/or guardian during care.

Initial presentation of the rotation

On the first day, the telemedicine service is introduced to residents, along with video guidance on the operation of the care system and its importance in today's medicine.

### Tele-propaedeutics

Bibliography is available for study with a synchronous online lecture presentation lasting one hour, addressing the particularities of the physical examination at distance. After all the steps are completed (explanatory video, completion of the Reaction Assessment and synchronous online tele-propaedeutics theoretical class), residents are authorized to perform tele-assistance under supervision.

### Tele-simulation

Two tele-simulation sessions were carried out during the rotation, with a simulated telemedicine care scenario followed by a debriefing. The first one aims to identify the process and technique of care in a situation in which a serious case needs to be referred to face-to-face care (respiratory distress in an infant). In the second, a case without need for is simulated, in which telemedicine fulfills its role of patient orientation and education (low-risk traumatic brain injury).

### Mini-CEX and immediate feedback

At the end of the three weeks of training (once a semester), the residents undergo a formative assessment of the acquisition of clinical skills, attitudes, and behaviors through a Mini-CEX applied by the medical team. In addition, after each service, immediate feedback was provided by the attending physician, emphasizing positive points and points of improvement for future consultations.

In order to better understand the residents' impression regarding the insertion of telemedicine in the curriculum, obtaining an understanding of their view on the subject and its importance, an evaluation of the reaction and evaluation of the resident's perception was carried out in the implementation of telemedicine care.

### Reaction assessment

In this study, the Reaction Assessment was developed by the researchers based on the Kirkpatrick model adapted to the objectives of our training.^12^^,^^13^ We evaluated the first level in Kirkpatrick's evaluation model. It was one of the first to propose an evaluation system for educational programs in the organizational aspect and became one of the most known and used.^12^ In 1959, the first version of this model established four levels for evaluating training, namely reaction, learning, behavior, and results.^13^

When applying the Reaction Assessment, we aim to evaluate the residents' impression of the insertion of telemedicine in the curriculum, obtaining an understanding of their view on the subject and its importance. Each level has its relevance and allows the program to be more effective and evident as it progresses. But also, more complex results to be interpreted. In the first level, the reaction of the participants is evaluated with the intention of improvements in the content, didactic material, instructors and professors, workload and location, in addition to also considering the participant's satisfaction.^13^

Residents answered the Reaction Assessment (Appendix 1), which was applied twice: once before the beginning of the training and once after the first round through the rotation. The questionnaire used was answered *via* Google Forms, using the Likert scale system, with a range of responses from totally agree, agree, indifferent, disagree and totally disagree.

### Assessment from the resident's perspective

At the end of the pediatric emergencies rotation, students answered a perception questionnaire, in which specific questions regarding telemedicine training were included:

- Classify telemedicine care (excellent, good, regular or poor).
- Answer if you felt assisted during the consultations (yes, no or sometimes).
- Answer if you think telemedicine teaching is relevant (yes, no or sometimes).
- Answer if the rotation contributed to your learning in telemedicine (yes, no or sometimes).
- Open comments.

### Statistical analysis

A descriptive analysis of the variables studied was performed with categorical variables presented in frequency and percentage and continuous variables presented in average (standard deviation). Responses to the Reaction Assessment were graded as 1- Strongly disagree, 2- Disagree, 3- Indifferent, 4- Agree, 5- Strongly agree.

The results of the Reaction Assessment before and after training were compared for each statement using the Wilcoxon test for paired groups. In all analyses, a significance level of 5% was adopted. SPSS 22.0 software will be used for the analyses.

## Results

During 2021, 40 residents underwent training in telemedicine during the pediatric emergencies rotation, of which 24 responded to the Reaction Assessment before performing the first consultation and to the same assessment at the end of the first 3 weeks of training and teleconsultations. The perception questionnaire was answered by the 40 residents.

**Fig. 2:**
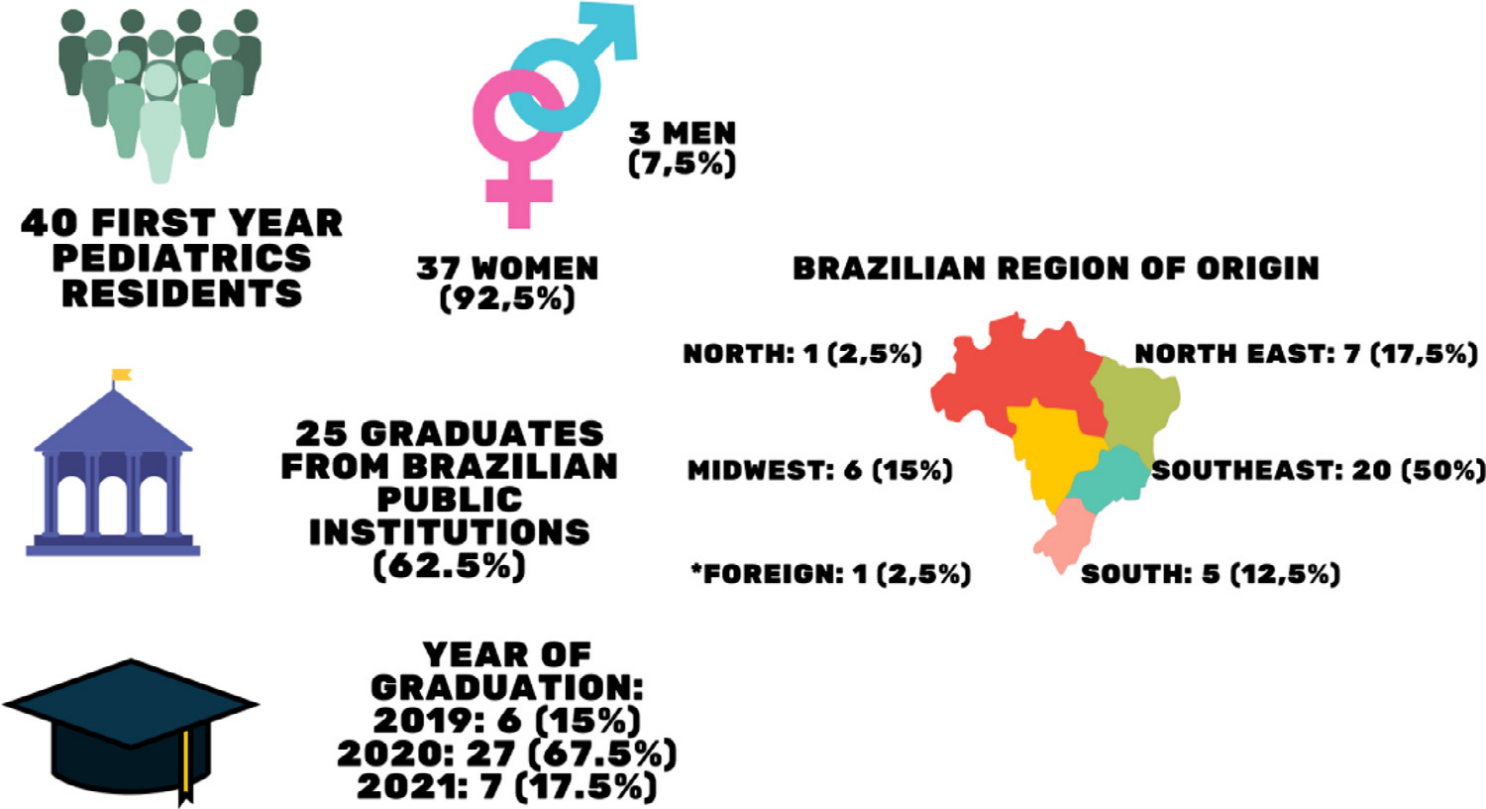
Sociodemographic characteristics.

The sociodemographic analysis (Fig. 2) showed that the majority of residents were female (self-reported) and the mean age was 25.3 years (σ = 0.93). More than half of them graduated from a public institution (25 residents ‒ 62.5%) less than 1 year ago (27 graduates in 2020 ‒ 67.5%). The origin of the residents varied among the regions of Brazil with half (50%) from the Southeast.

Between March and August 2021, the first semester of the residency, a total of 362 teleconsultations were carried out, an average of 9.05 (σ = 0.117) consultations/residents.

**Fig. 3:**
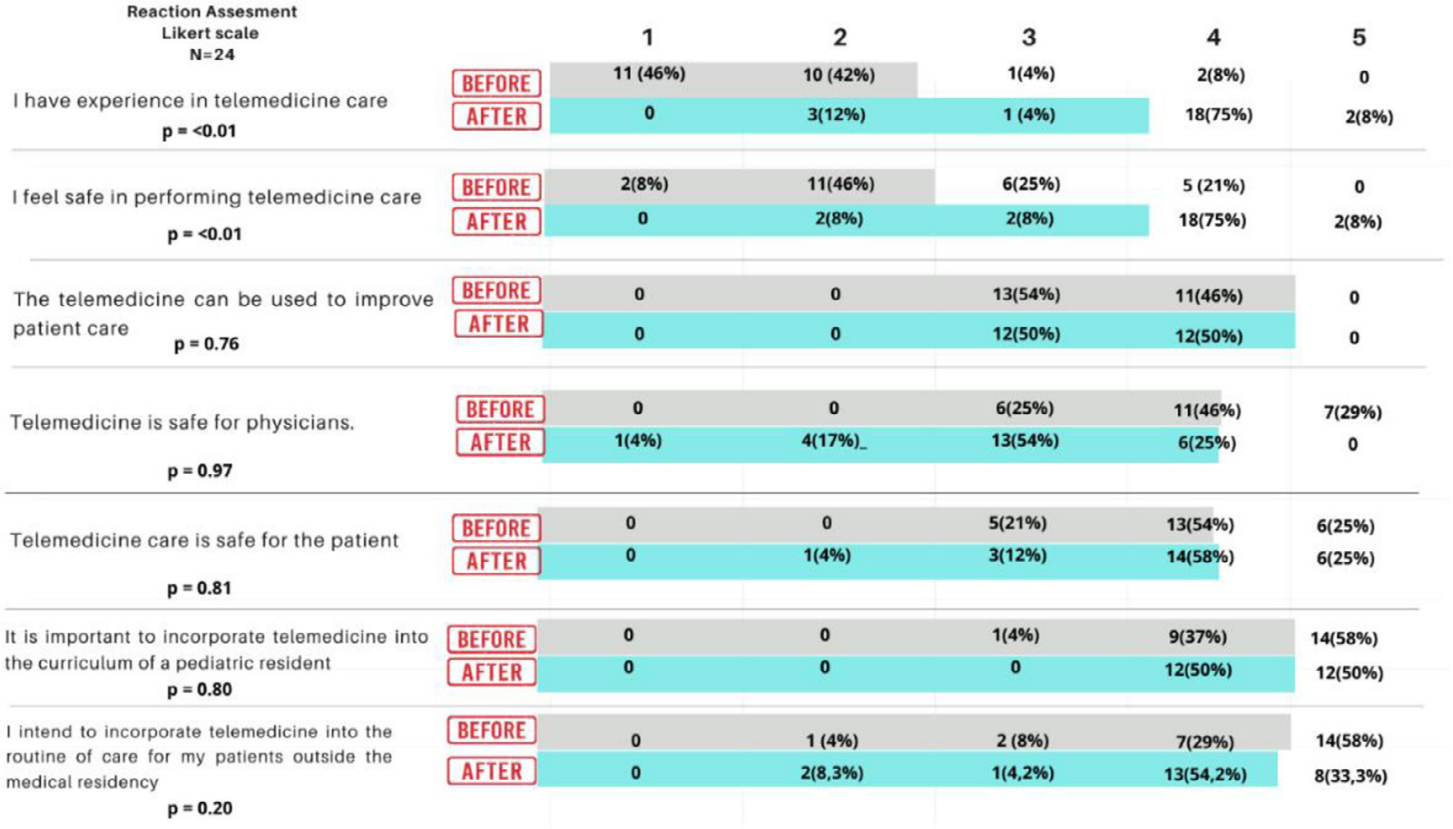
Reaction Assessment before and after the rotation. Likert Scale (1- Strongly disagree, 2- Disagree, 3- Indifferent, 4- Agree, 5- Strongly agree). The bar graph indicates the mean value of each response (see Table 1 for values).

The preliminary results corresponding to the first part of the training are shown in Fig. 3 and Table 1. There was a significant difference in the resident's perception of experience and safety after the initial training.

**Table 1:** Wilcoxon test for the reaction assessment.

	Before rotation (n = 24)	After rotation (n = 24)	p-value
I have experience in telemedicine care			<0.01
Median (range)	2 (1‒4)	4 (2‒5)	
Mean (SD)	2.58 (0.92)	3.8 (0.78)	
I feel safe in performing telemedicine care			<0.01
Median (range)	2 (1‒4)	4 (2‒5)	
Mean (SD)	2.6 (0.92)	3.8 (0.70)	
The telemedicine can be used to improve patient care			0.76
Median (range)	4 (4‒5)	4.5 (4‒5)	
Mean (SD)	4.5 (0.51)	4.5 (0.51)	
Telemedicine is safe for physicians.			0.97
Median (range)	4 (3‒5)	4 (2‒5)	
Mean (SD)	4.0 (0.75)	4.0 (0.78)	
Telemedicine care is safe for the patient			0.81
Median (range)	4 (3‒5)	4 (2‒5)	
Mean (SD)	4.0 (0.70)	4.0 (0.75)	
It is important to incorporate telemedicine into the curriculum of a pediatric resident			0.80
Median (range)	5 (3‒5)	4.5 (4‒5)	
Mean (SD)	4.5 (0.59)	4.5 (0.51)	
I intend to incorporate telemedicine into the routine of care for my patients outside the medical residency			0.20
Median (range)	5 (2‒5)	4 (2‒5)	
Mean (SD)	4.4 (0.83)	4.1 (0.85)	

SD, Standard Derivation.

In the evaluation from the resident's perspective, 3 (7.5%) classified the services as excellent, 24 (60%) as good and the other 13 (32.5%) as regular. The main complaints reported were technological difficulties (connection and image quality), repetitive care (reassessment of suspected cases of COVID-19) and little autonomy.

As for the relevance of training, 37 (92.5%) classified the teaching in tele-pediatrics as relevant and the other 3 residents as a partial relevance.

Considering assistance and supervision during training, 37 (92.5%) felt assisted in all sessions. Finally, 36 residents (90%) considered that the rotation contributed to their learning in telemedicine and the other 4 (10%) were indifferent to this issue.

## Discussion

We observed a remarkable impact on resident safety and their experience in telemedicine care after training. Despite the growth of telemedicine in the last 2 decades,^1^^,^^2^ it was only after the COVID-19 pandemic that virtual care spread.^14^ Considering that most residents graduated during the pandemic, the importance of telemedicine was already incorporated in the last year of medical graduation but with few training opportunities. This may justify their perception even before the training that telemedicine contributes to patient care, is safe for both physician and patient and should be incorporated into pediatrician teaching and routine.

The Reaction Assessment was applied during the internship routine and many of the participants did not respond to the first or second reaction questionnaire. This is certainly a limitation of our study. For this reason, the Reaction Assessment data corresponds only to the 24 complete responses. The perception questionnaire was answered by the 40 residents, as it was sent together with the final examination of the pediatric emergency internship, obtaining better adherence and response from all residents.

There are some studies in the literature like ours,^7^, ^8^, ^9^, ^10^ such as the Cleveland Clinic experience^10^ of a rotation in telemedicine for 148 Internal Medicine residents. It included training of preceptors and supervisors, interviews with residents before and after training, plus formative skills assessment (mini-CEX) and feedback after consultations. We have not found applications like this in pediatrics or pediatric emergencies.

Although the population of our study is smaller, it is possible to establish some connections. Both residents in our study and those at the Cleveland Clinic reported that they had little experience in telemedicine before rotation (86% vs. 76%). We also observed similarity in the intention to incorporate virtual care into their clinical practice, even before the rotation (83% vs. 70%) and in the safety of the resident physician's care after the rotation (80% vs. 83%) and a superiority in the contribution from rotation to telemedicine learning (90% vs. 76%). Immediate feedback after care and the mini-CEX were strengths highlighted in the perception questionnaire, which is like other similar experiences.^9^^,^^10^

Another interesting study in the United States evaluated the impact of the COVID-19 pandemic in pediatric residents training and curriculum.^15^ There were many losses in fields as procedural competence, outpatient clinical education and conferences. But their participation in telemedicine increased significantly during the pandemic, compared to prior in both inpatient and outpatient settings. Also, at least one-third reported being excluded from the care of patients with suspected or confirmed COVID-19. Telemedicine training could have provided residents with greater participation in caring for COVID-19 patients.

Yale University has also implemented a new virtual simulation program in response to the COVID-19 pandemic.^16^ Many students adhered to the elective program, and they have quickly adapted to the online scenarios, with positive changes in the communication pattern. It shows that there is genuine interest among students in telemedicine learning and that universities need to adapt, by creating a teaching model to be a part of the standard curriculum for medical students. Our study proposes a teaching model in telemedicine for pediatric residents, which includes virtual simulation, but also theoretical classes, reaction assessment, attendance of real pediatric patients, formative assessment (Mini-CEX) and others. It could be extended to medical students and residents of other specialties.

Referring to the educational objectives proposed in a competency-based curriculum,^11^ the skills most observed among resident physicians after training were professionalism, communication, collaboration and medical expertise, which are in line with the rotation proposal.

In the resident perception questionnaire applied after the rotation, the training in telemedicine received praises, arousing interest and confidence in attending through this modality. It can be seen as an opportunity for learning in virtual care and has the potential to contribute to the development of other important skills also for face-to-face care such as doctor-patient relationship, anamnesis and identification of severity.^9^^,^^10^ It was also mentioned the fact that it is a type of assistance that can be dynamic and resolute.

The PATMHO is pioneer in offering telemedicine in a public and exclusively pediatric tertiary service.^6^ The present study demonstrates an avant-garde telemedicine training initiative for pediatric residents, focusing on pediatric emergencies. Telemedicine is probably one of the legacies of the COVID-19 pandemic^14^^,^^17^ and we observed that the pediatric residents of our service already recognize the importance of learning more about virtual care. When answering the perception questionnaire, the resident physicians showed satisfaction with something so innovative and current.

Some divergences in the conduct of virtual care within the assistant medical team were highlighted by the residents. As it is a new modality, learning is constant and given the safety even among the most experienced, which would justify, in part, the little autonomy given to them. After the study, we intend to perform some consultations with remote medical assistance (another location and device). The medical physician will provide technical assistance and intervention only if necessary. To improve assistant equipment in virtual consultations, the institution recorded a course in digital health available to everyone on the “e-learning” platform.

Other studies that involve the telemedicine are interesting to mentioned. The RUTE Brazilian Network^18^ was designed as a tool in 2006 to help connecting health professionals, different medical practices and specializations, including pediatric healthcare all over the world. It shows us how useful the telemedicine could be, especially in big countries as Brazil to connect not only the doctor and patient, but all health professionals involved in patient´s care.

Technological barriers were observed by both the residents and those responsible for the patient. Poor connection, image quality and difficulty in accessing the system are some examples and evoke the need for investments in technology and digital inclusion of the Brazilian population.^19^^,^^20^

In addition to the mentioned limitations, we can highlight that this is a single-center study with a small sample (40 residents). Also, most consultations involved reassessments of face-to-face evaluations with similar complaints of respiratory problems during the pandemic.^6^ We considered that there were few cases/residents (approximately 9 per semester) due to the service being new and still in the initial years of implementation. Even so, residents considered that there was no impairment in learning.

## Conclusion

This study describes an innovative rotation proposal, including telemedicine in the curriculum of pediatric residents. Our preliminary results are encouraging, demonstrating the program's potential in training future pediatricians, developing skills for virtual and face-to-face care.

As next steps, we intend to assess other levels of Kirkpatrick, improve training and competence development, apply assessments of clinical skills, attitudes, and behaviors, in addition to evaluating the medium- and long-term impact of the rotation on the clinical practice of the future pediatrician.

## Funding

This research did not receive any specific grant from funding agencies in the public, commercial or not-for-profit sectors.

The authors are part of the coordination of the residency program in pediatric emergencies at Instituto da Criança e do Adolescente do Hospital das Clinicas da Universidade de São Paulo.

This study was part of the course conclusion work in the Specialization Course in Health Education (CEES) held at FMUSP.

## CRediT authorship contribution statement

**Rafael da Silva Giannasi Severini:** Methodology, Writing – review & editing, Supervision, Data curation. **Michelle Marcovici:** Supervision, Methodology, Writing – review & editing, Data curation. **Sylvia Costa Lima Farhat:** Data curation, Writing – review & editing. **Danielle Bivanco-Lima:** Data curation, Formal analysis, Supervision, Writing – review & editing. **Thomaz Bittencourt Couto:** Methodology, Writing – review & editing, Supervision, Data curation. **Ana Carolina Amarante:** Data curation. **Katharina Reichmann Rodrigues:** Data curation, Writing – review & editing. **Danielle Saad Nemer Bou Ghosn:** Methodology, Writing – review & editing, Supervision. **Cláudio Schvartsman:** Data curation, Formal analysis, Methodology, Writing – review & editing, Supervision.

## Conflicts of interest

The authors declare no conflicts of interest.

## Appendix Supplementary materials

Image, application 1
