# Supplementary material for: How to incorporate telemedicine in medical residency: A Brazilian experience in pediatric emergency
Source: Clinics (Sao Paulo). 2023 Feb 16;78:100162. doi: 10.1016/j.clinsp.2022.100162 (PMC9933318; doi:10.1016/j.clinsp.2022.100162)
Supplement: Supplementary file 1 [file mmc1.docx]

CLINICS-D-22-00194 – Supplementary Material

**Appendix 1** Reaction Assessment.

| **Answer the questions:** | | | | | |
| --- | --- | --- | --- | --- | --- |
| **NAME:** | | | | | |
|  | Strongly agree | Agree | Neutral | Disagree | Strongly disagree |
| I have experience in telemedicine care |  |  |  |  |  |
| I feel safe in performing telemedicine care |  |  |  |  |  |
| The telemedicine can be used to improve patient care |  |  |  |  |  |
| Telemedicine is safe for physicians. |  |  |  |  |  |
| Telemedicine care is safe for the patient |  |  |  |  |  |
| It is important to incorporate telemedicine into the curriculum of a pediatric resident |  |  |  |  |  |
| I intend to incorporate telemedicine into the routine of care for my patients outside the medical residency |  |  |  |  |  |
